# A 15 month experience with a primary care-based telemedicine screening program for diabetic retinopathy

**DOI:** 10.1186/s12886-021-01828-3

**Published:** 2021-02-04

**Authors:** James E. Benjamin, Justin Sun, Devin Cohen, Joseph Matz, Angela Barbera, Jeffrey Henderer, Lorrie Cheng, Julia Grachevskaya, Rajnikant Shah, Yi Zhang

**Affiliations:** 1 Department of Ophthalmology, Lewis Katz School of Medicine at Temple University, Philadelphia, PA USA; 2 Lewis Katz School of Medicine at Temple University, Philadelphia, PA USA

**Keywords:** Diabetes, Telemedicine, Teleophthalmology, Diabetic retinopathy, Fundus photography, Retinal screening

## Abstract

**Background:**

Using telemedicine for diabetic retinal screening is becoming popular especially amongst at-risk urban communities with poor access to care. The goal of the diabetic telemedicine project at Temple University Hospital is to improve cost-effective access to appropriate retinal care to those in need of close monitoring and/or treatment.

**Methods:**

This will be a retrospective review of 15 months of data from March 2016 to May 2017. We will investigate how many patients were screened, how interpretable the photographs were, how often the photographs generated a diagnosis of diabetic retinopathy (DR) based on the screening photo, and how many patients followed-up for an exam in the office, if indicated.

**Results:**

Six-hundred eighty-nine (689) digital retinal screening exams on 1377 eyes of diabetic patients were conducted in Temple’s primary care clinic. The majority of the photographs were read to have no retinopathy (755, 54.8%). Among all of the screening exams, 357 (51.8%) triggered a request for a referral to ophthalmology. Four-hundred forty-nine (449, 32.6%) of the photos were felt to be uninterpretable by the clinician. Referrals were meant to be requested for DR found in one or both eyes, inability to assess presence of retinopathy in one or both eyes, or for suspicion of a different ophthalmic diagnosis. Sixty-seven patients (9.7%) were suspected to have another ophthalmic condition based on other findings in the retinal photographs. Among the 34 patients that were successfully completed a referral visit to Temple ophthalmology, there was good concordance between the level of DR detected by their screening fundus photographs and visit diagnosis.

**Conclusions:**

Although a little more than half of the patients did not have diabetic eye disease, about half needed a referral to ophthalmology. However, only 9.5% of the referral-warranted exams actually received an eye exam. Mere identification of referral-warranted diabetic retinopathy and other ophthalmic conditions is not enough. A successful telemedicine screening program must close the communication gap between screening and diagnosis by reviewer to provide timely follow-up by eye care specialists.

## Background

Diabetes is the leading cause of blindness among middle-aged and elderly individuals in the United States (U.S.) [1]. Almost all type 1 diabetics and roughly 80% of type 2 diabetics will develop retinal damage after 20 years with the disease [2]. Treatments including photocoagulation therapy and anti-VEGF injections if administered early in the disease course have been shown to be 90% effective in preventing blindness [3].

Despite the efficacy of early detection and treatment, only about half of all patients with diabetes receive proper screening and under 40% of patients with a high risk of vision loss ever undergo treatment [4, 5]. A multitude of factors have been suggested to contribute to this deficiency in modern diabetic eye care including, but not limited to, transportation barriers, financial burden, lack of education, and poor patient-physician communication and understanding [6]. This results in widespread lack of patient insight as demonstrated by a study from the 2005–2008 National Health and Nutrition Examination Survey which concluded that 73% of patients with DR were unaware of their condition [7].

These barriers disproportionately affect minority and underserved communities where there are significantly higher rates of DR and associated vision loss. A study of Los Angeles inner-city minority residents demonstrated that these patients were 3.5 times more likely to present with advanced DR than patients in a more affluent, predominantly white setting [8]. Unfortunately, minorities are also less likely to have annual eye examinations with African American and Hispanic diabetics having a 32–49% annual eye examination rate compared to the average 63.3% rate determined by the Behavioral Risk Factor Surveillance System of all patients with self-reported diabetes [9].

Remote fundus photography is an accurate and practical screening modality that has been developed to address these healthcare disparities and improve DR screening rates [10]. While efficacious and broadly administered programs have been well established in the UK and Australia for decades, telemedical DR detection has only recently gained traction in the U.S. The Veteran Affairs and the Indian Health Service-Joslin Vision Network have introduced two of the earliest and most widespread programs, both of which have resulted in significantly improved screening and treatment rates for their respective patient cohorts, demonstrating the effectiveness of teleophthalmology in screening at-risk populations [7]. Pairing these increased screening events with diabetic education may also improve overall diabetes management as suggested by one study in which patients who underwent remote fundus photography and education decreased their hemoglobin A1c (HbA1c) by 1.61 points compared to those who received endocrinology evaluation alone [11].

While several studies have observed the impact of digital imaging on increased screening rates, there has been limited investigation on whether this improved screening leads to increased attendance at ophthalmology referral appointments, especially in urban communities which are often afflicted with inflated no-show rates. Therefore, our study aims to evaluate how often positive retinopathy screening exams resulted in a completed ophthalmology appointment among Temple University Hospital (TUH) patients, a predominantly African American, Hispanic and underserved population in northern Philadelphia.

## Methods

**Fig. 1:**
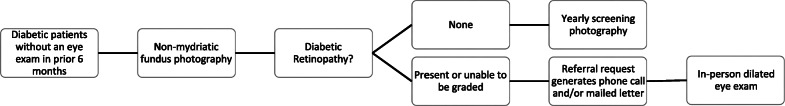
Flow Chart for Diabetic Retinopathy Telemedicine Screening

The study was a retrospective medical chart review of all patients aged 18 years and older who participated in the telemedicine retinal screening initiative at TUH from March 2016 to June 2017. Diabetic patients without a recent eye exam in the Temple electronic health record underwent bilateral non-mydriatic fundus photography with a Canon © CR-2 AF Digital Non-Mydriatic Fundus Camera (Canon Inc., Tokyo, Japan). One 45° field per eye of the posterior pole were captured by a trained nurse in the primary care doctor’s office and stored on Smart Care Doc (Telemed Ventures, LLC) an online electronic health record accessible only to select TUH employed ophthalmologists. The images were later accessed on a remote desktop computer where they were interpreted by a TUH board-certified optometrist or ophthalmologist with experience in the assessment of diabetic retinopathy from slit lamp biomicroscopy as well as digital fundus photography. A determination was made as to whether the patient should undergo further examination based on the appearance of the digital photograph. A referral was requested if there were any signs of diabetic retinopathy observed on the fundus photograph, if the image was uninterpretable, or if any other ophthalmologic conditions were identified. A phone call was made by TUH staff to arrange appointment. If patient could not be reached, a letter was mailed to for patient to make a specialist appointment (Fig. 1).

### Study population

Patients included in the screening program were ≥ 18 years old, had a diagnosis of diabetes mellitus (ICD10: E08.xxxx, E10.xxxx, E11.xxxx, E13.xxxx) at the time of their primary care visit, and had no recent (within one year) dilated fundus examination for diabetic retinopathy. Exclusion criteria included any subjects who were infants, minors, or prisoners.

### Data collection

Clinical data were abstracted from the electronic medical records by using a standardized data collection form. Training was provided and follow up meetings were scheduled to ensure consistent and accurate data collection and documentation. This study was approved by the Temple IRB. A waiver of HIPAA authorization was granted to improve the feasibility of this retrospective study. Collected patient information was stored on password protected Temple University Hospital computers within the Department of Ophthalmology, and backed up into an encrypted excel spreadsheet stored on a TUH Ophthalmology cloud-based folder to ensure no breach of patient confidentiality. Collected patient information was de-identified prior to data analysis to further ensure patient confidentiality.

Data included demographic data (date of birth, age at screening, sex, race), insurance carrier (Medicare, Medicaid, private, none), diabetes control method (diet, oral, insulin), and HgbA1c within 6 months of screening were collected. The date of fundus photography, date of fundus photo interpretation, fundus photograph quality of the right and left eyes (poor, fair, good, unable to be assessed, or unspecified), DR grade based on the International Classification of Diabetic Retinopathy (ICDR) system (no assessment due to poor photo quality, none, mild, moderate, severe, proliferative, or unspecified), and other ophthalmic diagnoses were recorded. Referral request and specialist appointments within 190 days of photo screening date (not made, made and showed, made and no showed) were also documented. At the specialist appointment, completion of a dilated fundus exam, presence of DR, grade of DR, and ultimate diagnosis were recorded.

### Statistical analysis

A chi-square analysis was performed on three different groups of diabetic management (diet-controlled, oral medication, and insulin) to grade statistical significance on the prevalence of DR. A similar, separate chi-square analysis was performed on three different A1c levels (4–7.9, 8.0–11.19, and > 12.0) on the prevalence of DR. A paired student’s t-test was used to discern a difference in A1c between the first and second screening.

## Results

Between 3/14/2016 and 5/26/2017, 689 digital retinal screening exams (1377 fundus photographs) were conducted in Temple’s primary care clinic. Six hundred sixty-three patients received one screening, while 26 patients received two screenings during this span of time. Of the 689 encounters, 404 (58.6%) women and the median age at the time of encounter was 59 years (range, 18–94 years). Five hundred forty-four (79.0%) screenings were performed on African Americans and 75 (10.9%) on Hispanics. The remaining 70 screenings (10.2%) were attended by Caucasians, unspecified or mixed-race patients, Asian/Pacific Islanders, and Native Americans. At the time of screening, 277 (40.2%) of 689 patients had a form of Medicare, 238 (34.5%) had a form of Medicaid, 152 (22.1%) had private insurance, and 20 patients (2.9%) had no insurance (Table 1). For comparison, Temple University Hospital’s North Philadelphia community has 464,455 residents, of which, 46% are African American, 30% are Hispanic and 18% are White. Fifty-three percent of this population is 50 or older, and 46% are covered by Medicaid with 40% covered by Medicare [12].

**Table 1:** Patient Demographics

Overall Demographics	
Number of Encounters	689.0
Female/Male	285/404 (58.6% female)
Mean age at screening (years)	59.3
Average HbA1c	8.1
Ethnicity	
African American	544 (79.0)
Hispanic	75 (10.9)
Caucasian	26 (3.8)
Asian/Pacific Islander	5 (0.7)
Indian	1 (0.1)
Other or Mixed	38 (5.5)
Insurance	
Medicare	277 (40.2)
Medicaid	238 (34.5)
Private	152 (22.1)
None	20 (2.9)
Medication Use	
Diet Only	116 (16.8)
Oral Medication Only	309 (44.8)
Insulin Dependent	264 (38.3)

*Data is reported as number (%)

**Fig. 2:**
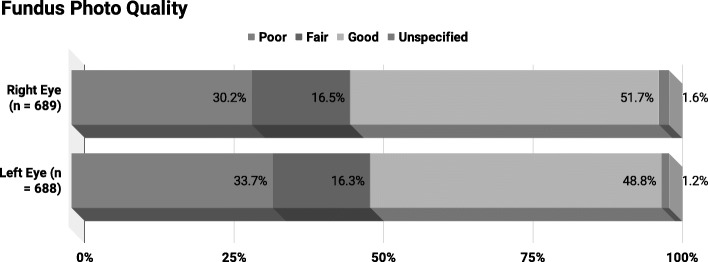
Screening Fundus Photo Quality

Figure 2 depicts the breakdown of fundus photograph quality in each eye. For the right eye, 356 (51.7%) of 689 photographs were specified as good quality, while 114 (16.5%) fair, 208 (30.2%) poor, and 11 (1.6%) unspecified either due to ocular condition or for unknown reason. A similar distribution of quality was noted for photographs of the left eye in which 336 (48.8%) of 688 photographs were specified as good, 112 (16.3%) were fair, 232 (33.7%) were poor, 8 images (1.2%) were unspecified, and 1 image was unavailable. The mean and median length of time between the screening visit and the fundus photo interpretation was 55.4 and 23 days, respectively (range, 0–418 days).

**Fig. 3:**
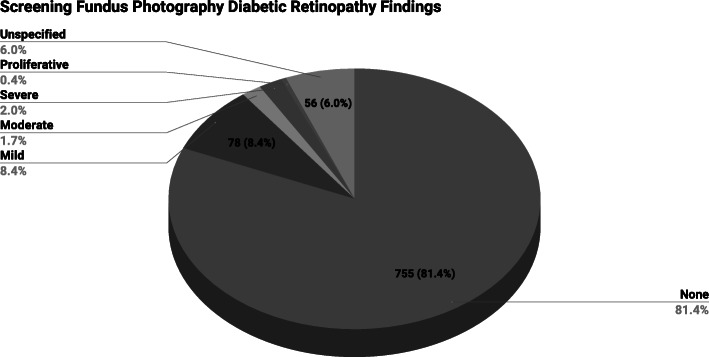
Screening Fundus Photography Diabetic Retinopathy Findings

We photographed 1377 eyes of 689 patients. Among them, 928 (67.4%) photos were gradable and 449 (32.6%) were unable to be assessed. Figure 3 displays the DR assessment of each fundus photograph that was able to be graded. Of these 928 graded images, 755 (81.4%) were read to have no retinopathy, 56 (6.0%) were read as DR with no modifier, 78 (8.4%) mild, 16 (1.7%) moderate, 19 (2.0%) severe, and 4 (0.4%) were graded as PDR.

When analyzing data in terms of patients, 343 (49.8%) of 689 patients were found to definitively have no DR in either eye based on fundus photography. One hundred-three patients (14.9%) were found to have some level of DR in at least one eye (70 (10.2%). Of the 103 patients with at least some DR in at least one eye, 33 (4.8%) patients had at least one image graded as ‘unspecified DR’. Of the 70 patients with specified grades of DR, 48 (68.6%) had mild, 9 (12.9%) had moderate, 11 (15.7%) had severe, and 2 (2.9%) had proliferative DR in one or both eyes.

**Fig. 4:**
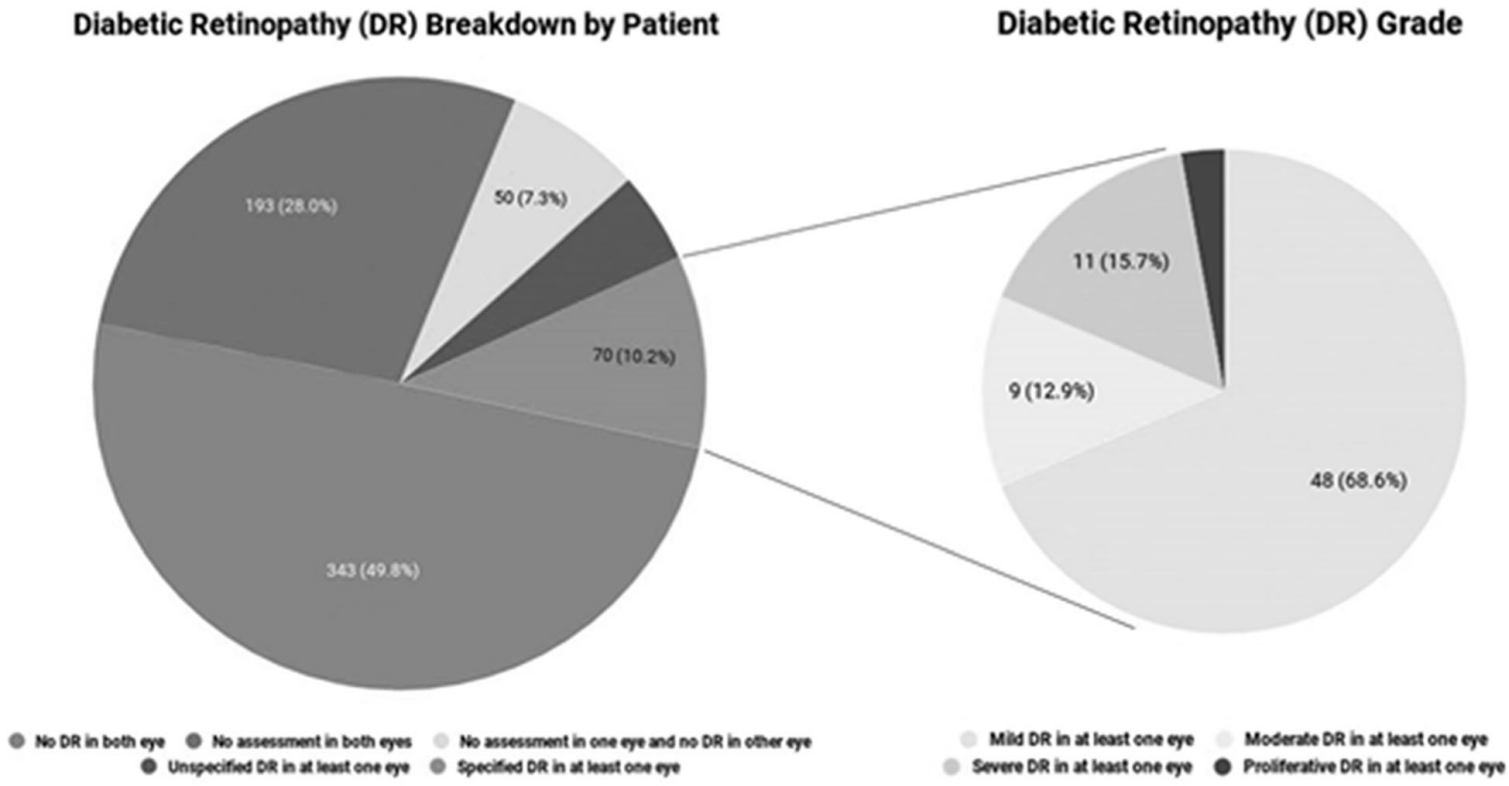
Diabetic Retinopathy Grade by Patient

Of the remaining 243 patients, as 193 (28.0% of 689 total) of them were unable to be assessed in both eyes and 50 (7.3% of 689 total) of them lacked DR in one eye but were unable to be assessed in the other (Fig. 4).

Sixty-seven (9.7%) of 689 patients were suspected to have another ophthalmic condition based on other findings in the retinal photographs. The most frequently encountered findings were those associated with glaucoma, hypertensive retinopathy such as vascular tortuosity, and macular or peripheral drusen.

**Fig. 5:**
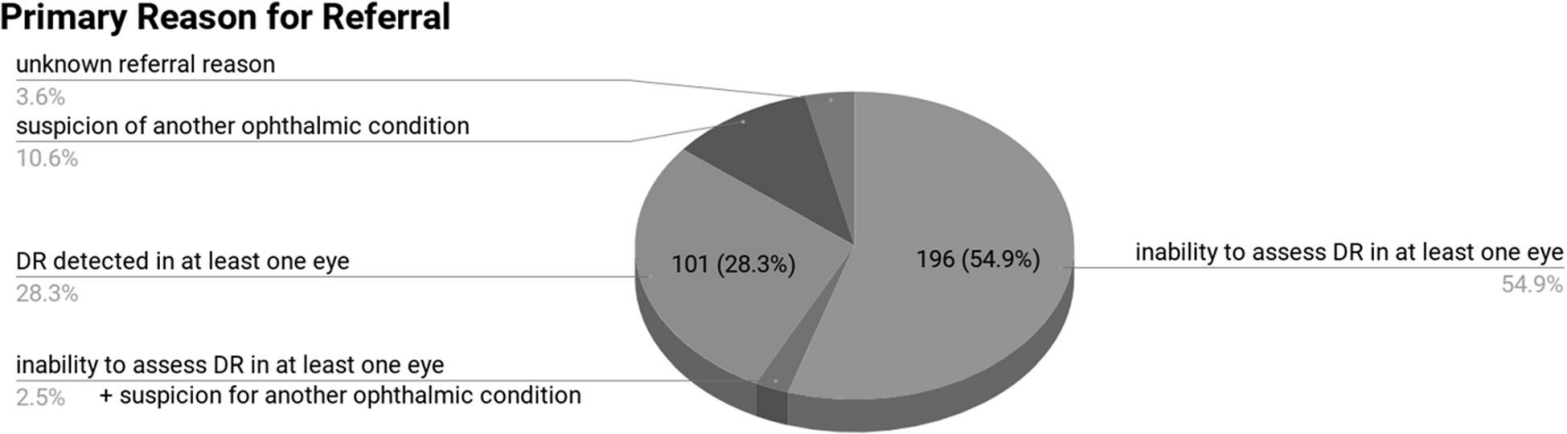
Primary Reason for Referral

Among the 689 screening exams, 357 (51.8%) resulted in a request for a referral to ophthalmology. Referrals were requested for DR found in one or both eyes, inability to assess presence of retinopathy in one or both eyes, or for suspicion of a different ophthalmic diagnosis. One hundred ninety-six (54.9%) of the 357 referrals resulted from an inability to assess DR in at least one eye, 101 (28.3%) were for some level of DR detected in at least one eye, 38 (10.6%) were for suspicion of another ophthalmic condition, Nine (2.5%) had at least one photograph that was unable to be assessed as well as suspicion for another condition, and 13 (3.6%) were referred in error since they had no suspicion of DR or another condition (Fig. 5). Of note, there were two instances of patients with detected DR or another ocular condition that did not result in a referral request.

**Fig. 6:**
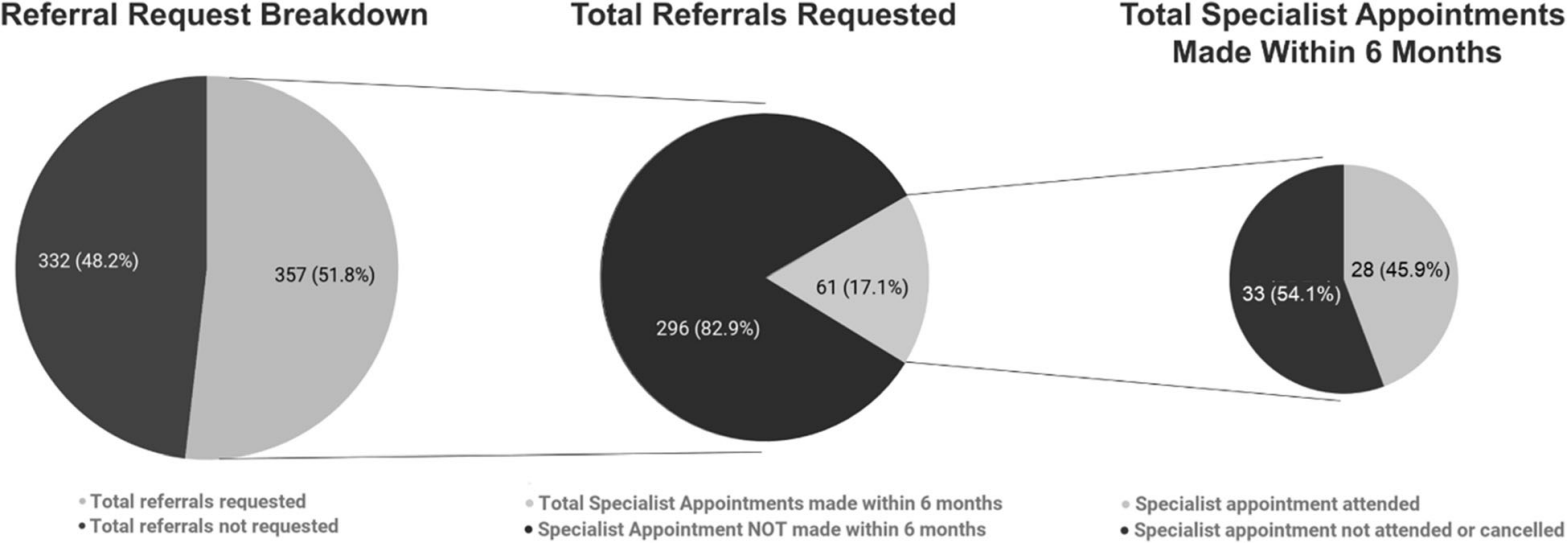
Patient Referral Request, Specialist Appointment Request, and Patient Attendance

We counted specialist appointments as being the result of the screening if the appointments were scheduled within a timeframe of 190 days from the digital fundus image interpretation date. Sixty (16.8%) of the 357 referral requests resulted in a scheduled appointment with the ophthalmology clinic. Two hundred ninety-six (82.9%) of the 357 referral requests did not result in an appointment being made. Among the 60 appointments, only 33 (55.0%) patients showed to the appointment while the other 27 (45.0%) appointments were either no-showed or cancelled (Fig. 6). The mean and median number of days between fundus photograph interpretation and scheduled ophthalmology appointment was 70.6 and 60.0 days, respectively (range 0–190 days).

Among the 33 patients that were successfully referred to Temple ophthalmology, there was good concordance between the level of DR detected by their screening fundus photographs and that diagnosed by specialist-performed dilated fundus exam (DFE). Eleven (32.4%) of the 33 patients were referred due to detected DR in at least one eye by the screening. Nine (82%) of these 11 were confirmed to have DR by on exam. Twenty-two (66.7%) of these 33 patients were referred, either due to inability to assess the photos or lack of findings. On exam, 17 (77.2%) of these 22 were confirmed to not have DR, while an assessment was unable to be made for one patient due to their visit focusing on their glaucoma as an undilated exam. Of the other five patients, one was found to have severe DR, two were found to have PDR, and two were diagnosed with unspecified DR. Among those patients in which a graded assessment was made by both the screening exam and the DFE, only one had significantly discordant findings – in this patient, the screening assessed mild retinopathy in both eyes and the DFE diagnosed PDR.

**Fig. 7:**
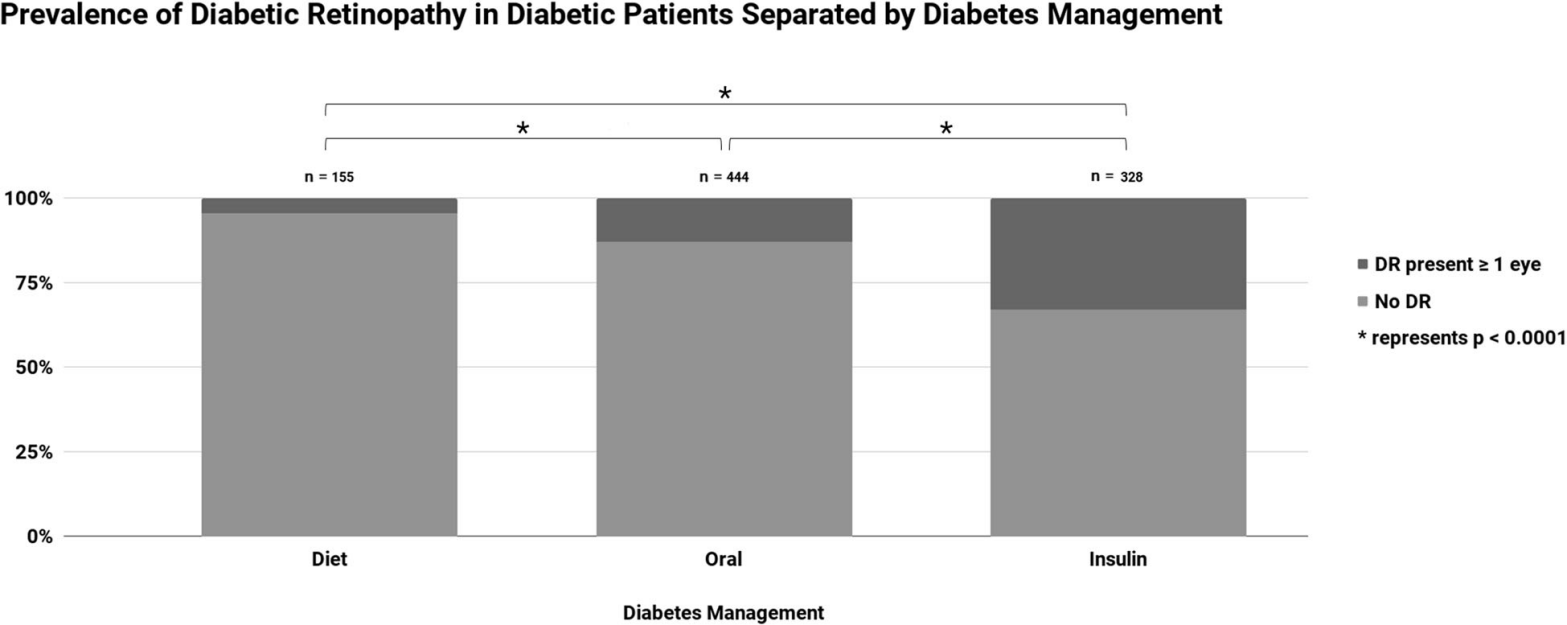
Diabetic Retinopathy Grade Breakdown by Diabetes Management

**Fig. 8:**
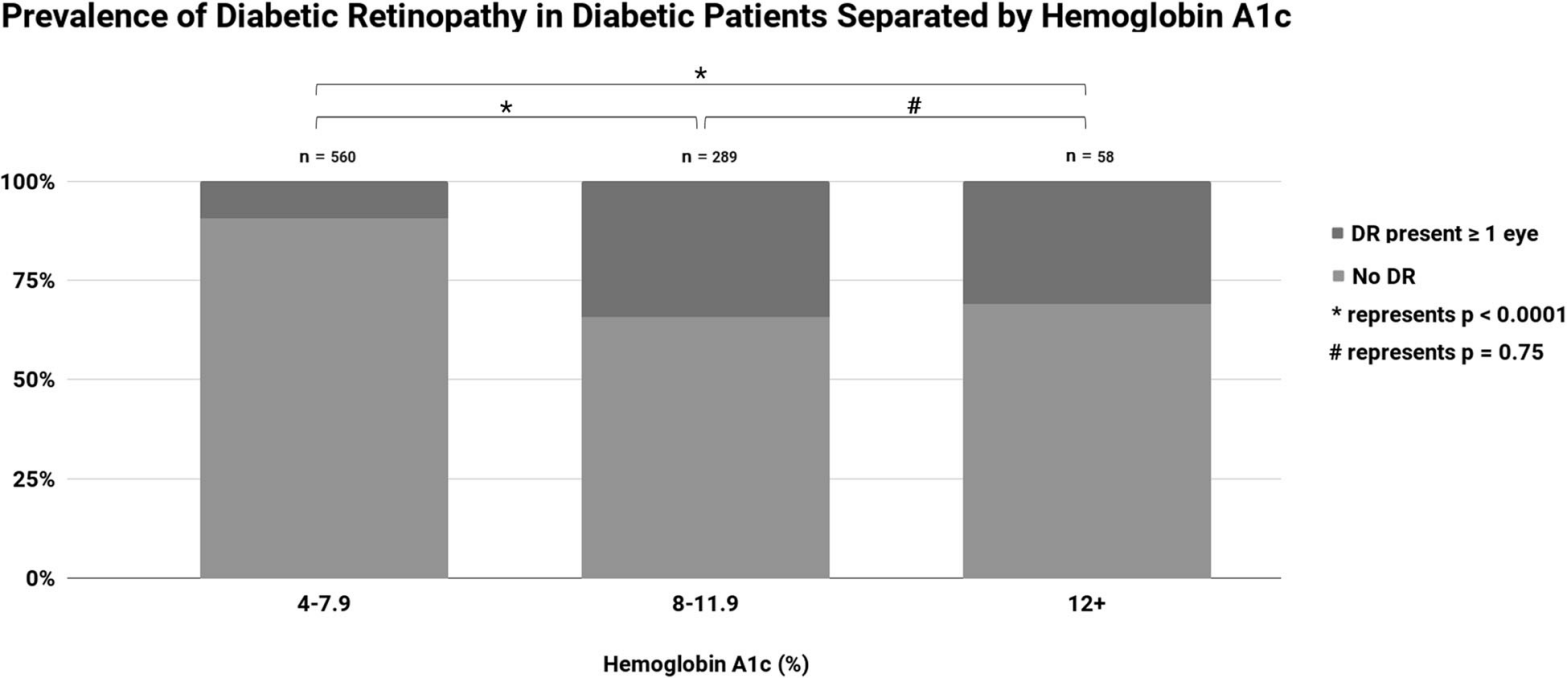
Diabetic Retinopathy Grade Breakdown by Hemoglobin A1c

We recorded the HgbA1c for 674 (97.8%) of 689 patients who had a documented value within 6 months of their respective screening dates. The average HgbA1c among these was 8.05% (range, 4.2–18.1%). Though not statistically significant, the 26 patients who had two screenings during our study period had an average A1c of 8.56% at the first visit and an average of 7.48% at the second, for an average reduction of 1.08% between visits (*p* = 0.054). At the time of screening, 116 of 689 (16.8%) patients were diet-controlled, 309 (44.8%) patients were being managed with only oral medication, and 264 (38.3%) were insulin-dependent (Table 1). More intensive diabetic therapy and higher A1c correlated with that of some degree of retinopathy in this patient cohort, demonstrated in Figs. 7 and 8, respectively. For those patients with gradable fundus photographs, 7/155 (4.5%) of the photos from diet-controlled diabetics displayed at least some level of DR compared to 57/444 (12.8%) and 108/328 (32.9%) of the photos of diabetics controlled with oral medications and insulin, respectively (*p* < 0.0001). Moreover, 53/560 (9.5%) of the images from patients with an HgbA1c between 4.0 and 7.9 showed DR as compared to 99/289 (34.3%) and 18/58 (31.0%) of those groups with values between 8.0–11.9 and > 12.0, respectively (*p* < 0.0001). The prevalence of diabetic retinopathy between the 8.0–11.9 and > 12.0 A1c groups was not statistically significant (*p* = 0.75) and was actually found to be higher in the lower A1c group (34.3% as opposed to 31.0%).

## Discussion

For those photos that could be graded, 18.6% of gradable photos had some degree of retinopathy. This number is consistent with other telemedicine screening programs in similar patient populations [9]. Of all the photos generated, only 67.4% were felt by the interpreting clinician to be readable. A similar telemedicine screening study with non-mydriatic fundus photography has shown an increased rate of readability of approximately 85% [13]. Our primary care staff operating the fundus camera need more training with non-mydriatic fundus photography as well as the ability to identify a good quality photograph. Taking multiple retina images of one eye or dilated fundus photography could increase the yield of gradable photographs but may increase visit duration and the level of training needed by the photographer. Dilating drops may also decrease the willingness of patients to be imaged. It is also unclear whether our primary care colleagues would be comfortable routinely dilating pupils with no eye care providers present. Furthermore, of the total referrals generated, 54.9% of them were due to the inability to assess for retinopathy in at least one eye. Poor quality photos could potentially generate many unnecessary referrals and can be avoided by improved training in non-mydriatic fundus photography.

There were 26 patients who had two separate screening encounters with screening photos and A1c tests. Though not statistically significant, the cohort decreased their average A1c by an average of 1.08% between visits. There are a myriad of other confounding factors leading to a reduction in A1c, but perhaps taking a diagnostic photo played a role in the motivation for these patients to better control their blood sugar collectively.

In terms of connecting patients with appropriate follow-up, less than 10% of referred patient actually completed an eye exam appointment. Specialist appointments were not being made at the same rate referrals were being generated. The single phone call to schedule an appointment with a letter generated for all unanswered phone calls is likely not enough. Perhaps there needs to be more of an effort by the screening program to reach out to these patients and possibly even establishing a designated telemedicine coordinator. Alternatively, there just may not be adequate public awareness of diabetic retinopathy in our patient population. Literature pamphlets of diabetic retinopathy depicting diabetic damage to the retina may highlight the importance of screening. Training nurses to appropriately educate patients how uncontrolled diabetes can lead to permanent vision impairment is critical. Liu et al. published a recent article on telemedicine where they conducted a series of interviews and found that a recommendation by a primary care doctor was one of the greatest motivators for getting the retinopathy screening photograph done [14]. This further highlights the value of awareness of eye health by both patients and the primary care community.

Additionally, we found that 67 patients (9.7%) of those screened had some other findings concerning for another ophthalmic condition, such as vein occlusions, glaucoma, or macular degeneration. Given the large number of unreliable photos, the numbers for other ophthalmic conditions are likely much higher in reality. In diabetic teleretinal screening literature, this figure can be as high as 50%, which is another potential benefit of screening [9].

The size of our cohort was a primary strength of the study as were able to assess 689 patients/1377 eyes over 15 months. In terms of limitations, as mentioned above, the poor ability to generate appointments from referrals truncates the effectiveness of a telemedicine screening program.

From a healthcare usage standpoint, identifying those patients at highest risk for eye disease will allow resources to be directed to that population. A majority of our photos were graded as completely normal (755/928 graded images, or 81.4%). It may be more beneficial to focus on a subset of patients that are particularly at high risk for development of retinopathy to increase the overall cost-effectiveness of screening. For example, it may be more efficacious in targeting photographs on patients with a certain number of years of diabetes, the intensity of their medication regimen, or a given A1c level. From our data, it appears that patients with an A1c > 8.0 and requiring insulin seem to have an increased the chance of retinopathy (*p* < 0.0001; see Figs. 5, 6). Furthermore, by identifying the patients who are at risk for vision loss earlier in the course of disease, we have the potential to decrease the costs associated with a visually impaired population [15]. In addition to increased accessibility to underserved populations telemedicine programs also offer a point of care solution during pandemic circumstances by helping triage patients in a timely manner.

Another potential difficulty we encountered was inconsistency in the time it took from the acquisition of the screening fundus photographs to interpretation. Some photos were interpreted as early as a few weeks from encounter, but some as late as a year. Artificial intelligence and neural networking is a rapidly advancing field and would tremendously increase the efficiency of image interpretation and reduce inter-reader variability. The IDX-DR (IDx LLC, Iowa City, IA, USA) is one such artificial intelligence diagnostic system that autonomously analyzes images of the retina for signs of diabetic retinopathy. This software has been studied in various formats of detection with impressive sensitivity and specificity results, but these parameters can change based on the magnitude of exam findings. In a 2019 article by Verbraak et al., vision-threatening diabetic retinopathy was detected with a sensitivity and specificity level of 100 and 97.8%, respectively. However, for moderate DR or worse, the sensitivity and specificity dropped to 79.4 and 93.8%, respectively, owing to the fact that more subtle changes need to be detected in those cases [16, 17]. Unfortunately, due to not referring any of the patients with normal retina images to be examined we are highly selecting against any false negatives. Our small sample of patients being referred as well as our biased population limits us on calculating a reliability sensitivity and specificity. Additionally, the EyeArt v1.2 (Eyenuk, Inc) is another diagnostic system that is able to align multiple retinal images from individual patients to evaluate new, persistent, and disappeared microaneurysms [18].

## Conclusions

Mere identification of referral-warranted diabetic retinopathy is not enough for a telemedicine screening program to succeed. Good quality fundus photography to avoid unnecessary referrals, patient education and prompt follow-up are central to a diabetic retinopathy telemedicine screening program. An effective and timely telemedicine screening program must take into consideration patient comfort, healthcare costs, and adequate time to care in order to prevent blindness in an appropriate timeline. By closing the communication gap between screening and diagnosis by reviewers, we can better serve our patient population and hope to reduce unnecessary blindness.

## Data Availability

The datasets used and/or analyzed during the current study are available from the corresponding author on reasonable request.

## Abbreviations

DR: Diabetic Retinopathy
VEGF: Vascular Endothelial Growth Factor
TUH: Temple University Hospital
PDR: Proliferative Diabetic Retinopathy
DFE: Dilated Fundus Exam
ICDR: International Classification of Diabetic Retinopathy
